# From Tissue to Transcriptome: A Systematic Review of Multi-Level Evidence for Immune Dysregulation in Atrial Fibrillation

**DOI:** 10.3390/jcm14207316

**Published:** 2025-10-16

**Authors:** Antonio da Silva Menezes Junior, Isabela Jubé Wastowski, Henrique Lima de Oliveira, Khissya Beatriz Alves de Lima, Silvia Marçal Botelho

**Affiliations:** 1 Internal Medicine, Faculty of Medicine, Federal University of Goiás, Goiânia 74690-900, Brazil; henrique.lima2@discente.ufg.br (H.L.d.O.); khissya_beatryz@discente.ufg.br (K.B.A.d.L.); 2 Medicine and Life School Science, Medicine School, Pontifical Catholic University of Goiás, Goiânia 74605-010, Brazil; smarcal@ufg.br; 3 Immunology Department, State University of Goiás, Goiânia 74845-090, Brazil; wastowski@gmail.com

**Keywords:** atrial fibrillation, immunity, T lymphocytes, inflammation, prognostic biomarker, Mendelian randomization

## Abstract

**Background:** Immune dysregulation has emerged as a central mechanism in atrial fibrillation (AF), with accumulating evidence implicating T-cell subsets, cellular senescence, checkpoint dysfunction, and inflammatory signaling. Although individual studies have provided important insights, a comprehensive synthesis across histological, mechanistic, prognostic, and genetic domains has been lacking. **Methods:** We systematically reviewed 16 studies published between 2009 and 2025, encompassing histological investigations, translational and mechanistic analyses, interventional cohorts, prognostic studies, and Mendelian randomization. Data on immune cell subsets, cytokines, signaling pathways, and clinical outcomes were extracted. Risk of bias was assessed using ROBINS-I and RoB 2, while certainty of evidence was graded using the GRADE framework. **Results:** Histological studies consistently demonstrated infiltration of atrial tissue by T lymphocytes and macrophages, with greater intensity in persistent and permanent AF, causally linked to atrial dilatation and fibrosis. Epicardial adipose tissue emerged as a key reservoir of tissue-resident memory T cells that promote IL-17- and IFN-γ-mediated fibroinflammatory remodeling. Mechanistic analyses highlighted CD8^+^PAR1^+^ cytotoxic T cells, PD-1/PD-L1 checkpoint disruption, and adipose–myocardial crosstalk as pivotal drivers of AF. Prognostic studies indicated that immune biomarkers provide incremental predictive value beyond conventional risk scores, while genetic evidence supported a causal role for immune dysregulation in AF susceptibility and progression. **Conclusions:** Across multiple levels of evidence, immune dysregulation is a primary determinant of AF development, progression, and outcomes. Integration of immune biomarkers into clinical practice may enhance risk stratification and inform the design of immune-targeted therapies for atrial fibrillation.

## 1. Introduction

Atrial fibrillation (AF) is the most sustained cardiac arrhythmia and is associated with considerable morbidity and mortality. Its prevalence is increasing with the aging population, lifestyle changes, and the increasing burden of cardiometabolic comorbidities [1,2]. AF is a significant risk factor for ischemic stroke, heart failure, cognitive decline, and premature death and represents a considerable proportion of healthcare expenditure [3,4]. Although catheter ablation and pharmacological treatment have advanced rhythm control and stroke prevention, recurrence rates remain high, indicating the need to further elucidate the mechanisms of AF initiation and perpetuation [5,6].

The pathophysiology of AF has traditionally been attributed to structural and electrical remodeling as well as atrial fibrosis. Nevertheless, recent evidence has indicated that inflammation and immune dysregulation play central roles in the genesis and perpetuation of arrhythmogenic substrate [7,8]. Experimental studies, as well as translational studies, have demonstrated that the NLRP3 inflammasome, cytokine networks, and oxidative stress contribute to atrial remodeling and conduction heterogeneity [9,10]. Recent clinical profiles also suggest that systemic inflammation, as reflected by circulating biomarkers including high-sensitivity C-reactive protein, interleukin-6, and tumor necrosis factor-alpha, is associated with the onset of AF arrhythmia, recurrence after ablation, and major adverse cardiovascular events [11,12]. These differences suggest that AF is an immune-mediated disease.

New research has highlighted the involvement of the adaptive immune system, T lymphocyte subsets, immune senescence, and malfunction of checkpoints. Recent reviews have highlighted that disturbed CD4/CD8 ratios, accumulation of senescent CD8+ T cells, and compromised regulatory T cell function may provide a connection between AF, thromboembolic complications, and adverse remodeling [13,14], such as cardiovascular aging and AF by Immunosenescence. Chronic low-grade inflammation and the expansion of dysfunctional lymphocytes are involved in cardiovascular aging and the progression of AF-related conditions [15]. Finally, the Epicardial adipose tissue (EAT) has been identified as an immunologically active compartment that generates proinflammatory mediators and directly interacts with the atrial myocardium, promoting fibrosis and arrhythmogenesis [16]. All these observations underscore a change in perspective: AF, initially considered a purely electrical phenomenon, has evolved into a systemic, tissue-specific inflammatory process.

Nonetheless, the literature is fragmented and inconclusive, primarily due to small sample sizes, single-center studies, and heterogeneity in immune phenotyping, which limit the generalizability of findings. The prognostic significance of immune biomarkers remains a topic of controversy, with conflicting results reported across various cohorts. Few studies have combined mechanistic, therapeutic, and genetic data. Current reviews have been predominantly narrative, and evidence has not been synthesized across these study types, including histological, translational, prognostic, and causal inference studies. The present systematic review was conducted to provide a comprehensive summary of T cell-mediated immunity, regulatory mechanisms, and immune-related biomarkers in AF and to minimize the lack of a theoretical basis or poor practical significance of these immune disorders in AF in all aspects, including their role in AF pathogenesis, prognosis, and therapeutic implications.

## 2. Materials and Methods

This systematic review was conducted in accordance with the “Preferred Reporting Items for Systematic Reviews and Meta-Analyses” (PRISMA) guidelines and followed the methodology framework established by the Cochrane Collaboration. The protocol for this review was prospectively registered with PROSPERO (ID: CRD420251038380), as seen in Supplementary Methods S1 and S2.

### 2.1. Eligibility Criteria

We included original studies that investigated the role of immune cells, particularly T lymphocyte subsets and related inflammatory pathways, in the pathogenesis, prognosis, and management of AF. Eligible designs included histological studies analyzing atrial tissue, mechanistic translational investigations, observational cohorts, randomized controlled trials, and Mendelian randomization studies. Adult patients with AF of any type (paroxysmal, persistent, or permanent) and control populations with sinus rhythm were considered. We included studies reporting outcomes related to atrial immune infiltration, immune checkpoint regulation, circulating immune biomarkers (e.g., CD4/CD8 ratio and senescent or regulatory subsets), immune responses to ablation, and genetic associations between immune traits and AF.

There were no restrictions on geographic region or setting, and both surgical cohorts and community- or hospital-based samples were eligible. Only peer-reviewed articles published in English between the years 2000 and 2025 were included in the analysis. Conference abstracts, reviews, editorials, and case reports were also excluded. Studies were excluded if they did not directly assess immune parameters or if AF was only reported as a secondary or incidental finding without specific immune-related outcomes. Animal and purely experimental laboratory studies without human tissue or clinical data were excluded. This approach enabled us to capture a broad, yet thematically consistent, body of evidence spanning local histological observations, mechanistic immune pathways, procedural immunomodulation, clinical prognostic biomarkers, and causal genetic analyses.

### 2.2. Search Strategy and Data Extraction

A comprehensive literature search was performed to identify studies that have evaluated the roles of T lymphocyte subsets, inflammatory pathways, and immune biomarkers in atrial fibrillation (AF). We systematically searched PubMed/MEDLINE, Embase, and the Cochrane Library from their inception to August 2025 using combinations of controlled vocabulary (MeSH, Emtree) and free-text terms. The main search blocks included population terms such as “atrial fibrillation,” “arrhythmia,” and “cardiac arrhythmia”; exposure/biomarker terms such as “T lymphocyte,” “T cell,” “CD4,” “CD8,” “regulatory T cell,” “senescent T cell,” “tissue-resident memory T cell,” “immune checkpoint,” “PD-1,” “PD-L1,” “inflammation,” “immune biomarkers,” and “epicardial adipose tissue”; and genetic/causal terms such as “Mendelian randomization,” “GWAS,” and “genetic association”. Boolean operators (“AND,” “OR”) were used to combine terms, and filters were applied to restrict the results to human studies and English-language publications. Reference lists of the included articles and relevant reviews were manually screened to identify additional eligible studies [16]. The complete PubMed search strategy is provided in the Supplementary Materials, and analogous strategies have been adapted for other databases. Two reviewers independently [A.S.M.J. and S.M.B] screened the titles and abstracts for relevance, followed by a full-text assessment of potentially eligible studies. Discrepancies were resolved by consensus or consultation with a third reviewer [I.J.W]. Data were extracted using a standardized data collection form designed a priori.

### 2.3. Endpoints and Subanalyses

Extracted information included study characteristics (first author, year, journal, country, study design, sample size, and population characteristics [age, sex, AF type, and control group]). Immune focus: specific cell subsets (e.g., CD3+, CD4+, CD8+, senescent, TRM, Tregs, and Bregs), molecular pathways (e.g., Protease-activated receptor-1 (PAR1) and PD-1/PD-L1), biomarkers, or genetic determinants. Methodology: Type of analysis (histology, immunohistochemistry, flow cytometry, cytokine assays, GWAS, and Mendelian randomization). Outcomes: Immune infiltration, fibrosis, atrial remodeling, AF incidence, recurrence, thromboembolic events, stroke, heart failure, cardiovascular mortality, and major adverse cardiovascular events (MACE). The effect estimates included odds ratios, hazard ratios, regression coefficients, and correlations, along with 95% confidence intervals (CIs) and *p*-values, where available. ROBINS-I assessed risk of bias for observational studies, the Cochrane tool was used for randomized trials, and GRADE was used for certainty of evidence across outcomes. The data were cross-checked for accuracy, and the most comprehensive dataset was used when multiple reports were derived from the same cohort.

### 2.4. Quality Assessment

The methodological quality of the included studies was assessed using the ROBINS-I for observational designs, the Cochrane risk-of-bias for randomized controlled trials, and the GRADE framework to evaluate the overall certainty of evidence across outcomes [17,18]. The GRADE criteria (risk of bias, inconsistency, indirectness, imprecision, and publication bias) were applied to rate the certainty of the evidence as high, moderate, low, or very low. For non-randomized studies, the evidence initially started at “low” and was upgraded or downgraded based on the effect size, dose–response relationships, and methodological limitations.

### 2.5. Statistical Analysis

This systematic review was performed in accordance with the Cochrane Collaboration and the Preferred Reporting Items for Systematic Reviews and Meta-Analysis (PRISMA) statement guidelines [17]. Threshold for significance: Across the included studies, *p* < 0.05 was considered statistically significant. Where reported, adjusted *p*-values were highlighted; effect sizes: results were expressed as odds ratios (OR), hazard ratios (HR), or regression coefficients with 95% confidence intervals (CI); correlation analyses: Pearson or Spearman coefficients (*r*) were reported with *p*-values; statistical significance was generally defined as *p* < 0.05. GRADE certainty: Evidence certainty was graded as high, moderate, low, or very low, reflecting confidence in the effect estimates rather than statistical significance alone.

## 3. Results

### 3.1. Study Selection and Characteristics

**Figure 1:**
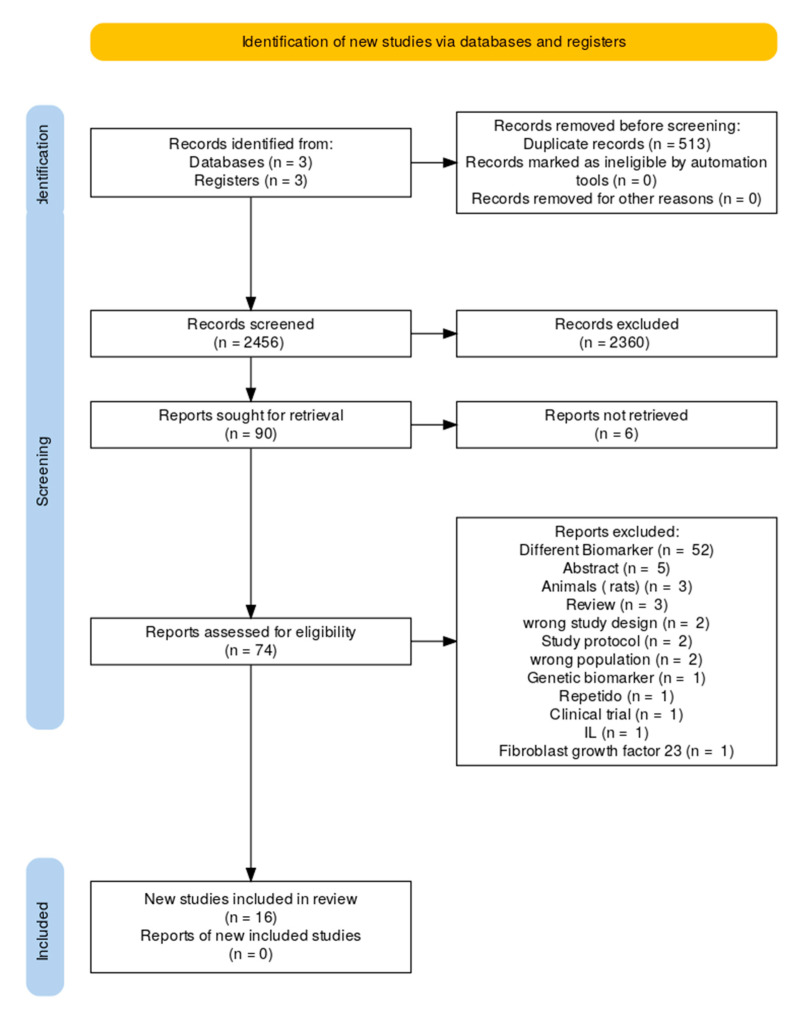
Flow chart of study selection.

**Figure 2:**
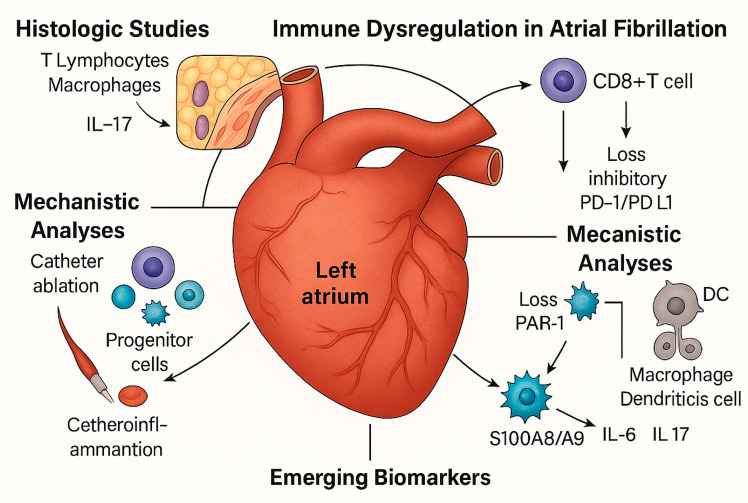
**Central Illustration**. Immune dysregulation is a central feature of atrial fibrillation (AF) across multiple levels of evidence. Histological studies revealed an increased infiltration of T lymphocytes and macrophages within the atrial myocardium. At the same time, epicardial adipose tissue serves as a reservoir of tissue-resident memory T cells that produce IL-17 and IFN-γ. Mechanistic analyses highlighted CD8+ T cell activation via PAR1 signaling and the loss of inhibitory PD-1/PD-L1 checkpoints. Interventional data have shown that catheter ablation mobilizes progenitor cells and modulates thromboinflammatory profiles. Clinical cohorts have identified circulating immune signatures, including CD4/CD8 ratio, senescent CD8+ cells, and regulatory subsets, as independent prognostic markers. Finally, genetic evidence confirmed the causal role of neutrophils, basophils, and CD4+ T cells, establishing an immune imbalance as integral to AF pathogenesis and outcomes. Source: the authors.

Sixteen studies were eligible [19,20,21,22,23,24,25,26,27,28,29,30,31,32,33,34]. These included histological (*n* = 4), translational mechanistic (*n* = 4), interventional cohort (*n* = 3), prognostic observational (*n* = 4), and genetic causal studies (*n* = 1), as seen in Figure 1. They encompass a range from small surgical tissue series to large population-based datasets and Mendelian randomization analyses involving more than 1 million people. The investigated immune elements involved the T lymphocytes (CD3, CD4, CD8), regulatory T cell subsets (Tregs, Bregs, DNTs), senescent cells (CD28−CD57+), progenitor cells (CD34+), immune checkpoints (PD-1/PD-L1), tissue-resident memory T cells, platelet–inflammatory biomarkers, granulocytes, and genetically determined cell counts. The reproducibility of the results across different approaches supports the evidence that immune dysregulation is a hallmark of AF, as shown in Figure 2 (Central Illustration).

### 3.2. Evidence of Immune Infiltration in Histology

Early histological studies revealed that the atrial tissue of patients showed greater infiltration by inflammatory cells than that of SR controls. Orgukova et al. [19] found an increase in colocalized T lymphocytes and macrophages within fibrotic areas, confirming inflammation as a structural correlate of remodeling. Smorodinova et al. [22] documented that the numbers of CD3+ T cells and CD68+ macrophages/dendritic cells preferentially increased in the left atrium of patients with AF, independent of systemic inflammation. Hohmann et al. [23] demonstrated that immune infiltration follows the natural history of AF, with a peak in persistent AF and a reduction in permanent AF, supporting the notion that inflammation is most active in the early phase of the disease, followed by an increase in fibrous tissue. Yamashita et al. [24] demonstrated a correlation between left atrial enlargement and increased fibrosis, as well as higher numbers of CD3+ T cells and CD68+ macrophages, further highlighting the interplay between structural and immune remodeling. Recently, Vyas et al. [25] described EAT as a site of TRM accumulation. In AF patients, cloned TRM populations migrated into the atrium, secreting IFN-γ and IL-17, and induced fibro-inflammatory changes in co-cultured cardiomyocytes. Spatial transcriptomics revealed enrichment of inflammatory signatures in the EATrial border zone. Together, these studies provide histological evidence that AF is characterized by rhythm- and size-dependent immune infiltration and that EAT represents a newly identified site for the localization of pathologic T cells (Table 1 and Table 2).

### 3.3. Mechanistic Routes in AF Immunopathology

The inflammatory processes can result in a substantial antigenic load, which, through molecular interaction between antigens and cardiac proteins, may induce systemic autoimmune activation against the myocardium or perpetuate an ongoing autoimmune reaction. Beyond descriptive histology, recent mechanistic studies have explored the immune pathways linking activation to atrial remodeling and thromboinflammation.

Friebel et al. [26] identified that cytotoxic CD8+ T cells expressing protease-activated receptor 1 (PAR1) were upregulated in patients with first-diagnosed AF. These cells are associated with atrial fibrosis and dysfunction, and their activation is suppressed by anticoagulation, indicating a connection between coagulation and immunity. Chang et al. [32] recently analyzed checkpoint regulation and found that PD-1 on CD4+ T cells and PD-L1 on myeloid dendritic cells were significantly downregulated in patients with AF. This dysregulation led to hyperactivated T cells, higher levels of cytokines (IL-2, IL-6, IL-10, IFN-γ), and an increased risk of stroke.

Vyas et al. [25] also provided mechanistic insights, revealing that EAT-derived TRM cells not only infiltrate the atrium but also disrupt calcium handling and initiate profibrotic signaling in atrial cardiomyocytes. These findings contribute to a better understanding of T cell immune checkpoint dysregulation (TICD) and its implications for AF. Together, these studies highlight that atrial fibrillation is not only an electrophysiological disorder but also an immunologically active condition involving multiple, interlinked immune pathways.

### 3.4. Interventional Evidence: Ablation/Immune Modulatory Therapies

Catheter ablation modifies immune pathways. Kim et al. [27] demonstrated that radiofrequency ablation mobilized CD34+ progenitor cells via an MMP-9/GROβ-related pathway, with mobilization associated with ablation time and the extent of myocardial injury, implicating a reparative immune axis.

Waleed et al. [28] conducted a randomized controlled trial of CB and RF ablations. Both were found to acutely elevate platelets and markers of inflammation. However, with CB ablation, there was less early platelet activation and a beneficial, sustained reduction in platelet biomarkers among patients who remained in sinus rhythm. The inflammatory indicators returned to the normal range in both groups after follow-up. These results highlight the fact that ablation modalities vary in their immunological impact, implying that changes in biomarker trends may help guide procedural selection.

### 3.5. Prognostic Immune Biomarkers in Patient Cohorts

Peripheral immune profiles have emerged as strong prognostic markers in atrial fibrillation. Stegbauer et al. [29] demonstrated that senescent CD8+ T cells (CD28^−^CD57^+^) independently predicted adverse outcomes, including stroke, heart failure hospitalization, and cardiovascular death, particularly in patients stratified by CHA_2_DS_2_-VASc score. Zhou et al. [30] found that a low CD4/CD8 ratio was independently associated with an increased incidence of AF and worse cardiovascular outcomes, thereby enhancing risk stratification beyond conventional clinical scores. In contrast, You et al. [31], analyzing the largest cohort to date (45,000 hospitalized patients), identified a high CD4/CD8 ratio as a predictor of similar adverse events. This apparent discrepancy may reflect differences in study populations: Zhou et al. [30] examined stable outpatients with coronary artery disease, whereas You et al. [31] evaluated a more complex, multimorbid inpatient cohort. Despite opposing directional associations, both studies underscore the prognostic value of CD4/CD8 ratio imbalance in AF.

Li et al. [33] found that, in stroke patients, various regulatory immune subpopulations—including CD4+ and CD8+ regulatory T cells, B regulatory cells (Bregs), and double-negative T cells (DNTs) dynamically predicted neurological outcomes, weighing the prognostic relevance of immune regulation in cardiovascular disease. Similarly, Vyas et al. [25] reported that elevated cytokine levels and a higher systemic inflammation score were associated with increased stroke risk in patients with atrial fibrillation. Collectively, these findings support the role of distinct immune profiles—including T cell senescence, CD4/CD8 ratio imbalances, regulatory subsets, and proinflammatory cytokine signatures—as independent predictors of adverse outcomes in AF.

Several studies have highlighted the prognostic value of immune biomarkers in atrial fibrillation (AF). Duygu et al. [20] reported that soluble CD40 ligand (sCD40L) was significantly elevated in patients with chronic nonvalvular AF who developed spontaneous contrast, left atrial thrombus, or embolic events—supporting its role as a marker of thromboembolic risk. Similarly, Ogurkova et al. [19] found that upregulation of the CD40/CD40L axis was associated with thrombotic complications, reinforcing thromboinflammatory pathways in AF.

Regarding T cell phenotypes, Sulzgruber et al. [21] showed that CD4+CD28-T cells were elevated in patients with AF and chronic heart failure and independently predicted cardiovascular mortality, alongside a reduction in regulatory T cells (Tregs). Stegbauer et al. [29] identified senescent CD8+ T cells (CD28-CD57+) as predictors of stroke, heart failure hospitalization, and cardiovascular death, emphasizing the role of immunosenescence in AF progression.

Zhou et al. [30] and You et al. [31] reported that imbalances in the CD4/CD8 ratio independently predicted adverse cardiovascular outcomes, although with divergent directional associations—likely reflecting differences in study populations. In a systems-level approach, Xiao et al. [36] used integrative bioinformatics to identify reduced Treg levels, increased myeloid cell infiltration, and activation of the calcineurin–NFAT signaling pathway as molecular signatures of persistent AF. These findings also suggested novel therapeutic targets, including miR-34a-5p, RCAN1, and PPP3R1. Collectively, these studies reinforce the relevance of immune dysregulation—particularly thromboinflammatory markers, immunosenescence, and T cell imbalance—as key predictors of adverse outcomes in AF, as shown in Table 3.

### 3.6. Genetic Evidence for Causality

Feng et al. [34] employed Mendelian randomization to investigate the causal relationship between immune cell profiles and AF. Using data from a genome-wide association study (GWAS) involving over one million individuals of European descent, they found that genetically determined increases in neutrophil, basophil, and CD4^+^ T cell counts were significantly associated with a higher risk of AF. Conversely, elevated levels of natural killer (NK) cells and total lymphocytes conferred a protective effect. These results provide some of the most substantial evidence to date that immune dysregulation is not merely an epiphenomenon but is likely causally implicated in the pathogenesis of AF.

### 3.7. Integrated Interpretation

Across these sixteen studies, the pattern was clear. Histological analysis demonstrated that AF atria are infiltrated by T cells and macrophages, the extent of which is related to rhythm, atrial size, and the presence of adjacent epicardial adipose tissue reservoirs. Mechanistic analyses identified the following immune axes: coagulation-associated CD8+PAR1+ cells, PD-1/PD-L1 checkpoint perturbation, and adipose-resident TRM cells, which couple immunity with fibrosis, remodeling, and thromboinflammation. Interventional testing has shown that ablation procedures are associated with progenitor mobilization and the regulation of thromboinflammatory biomarkers, suggesting that the treatment can exert an immune stimulus. Prognostic cohorts have demonstrated that peripheral immune signatures, including CD4/CD8 ratio, senescent CD8+ T cells, and regulatory subsets, independently predict inferior outcomes. Lastly, genetic analysis supports a cause-and-effect relationship, implicating neutrophils, basophils, and CD4+ T cells as predisposing factors and NK cells as protective factors.

**Figure 3:**
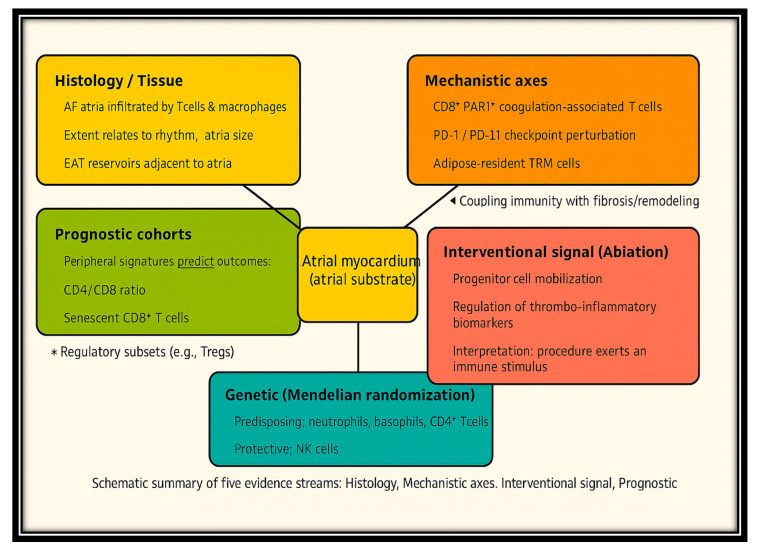
**Integration of Histologic, Mechanistic, Genetic, and Clinical Evidence for Immune Dysregulation in AF.** Legend: Cross-evidence synthesis of immune dysregulation in atrial fibrillation (AF). Five evidence streams—histology/tissue, mechanistic axes, interventional signal (ablation), prognostic cohorts, and genetic (Mendelian randomization)—converge on the atrial myocardium as the central substrate. Arrows highlight how immune mechanisms contribute to atrial remodeling, thromboinflammation, and AF progression.

The robustness of immune involvement across different designs, populations, and methods is remarkable. While histological and operative cohorts are subject to particularly high-risk confounding and selection bias, and prognostic studies vary in the directionality of specific ratios, a plethora of evidence supports the consensus that immune dysfunction is central to the induction, maintenance, progression, and clinical manifestations of AF, as shown in Figure 3.

### 3.8. Assessment of the Quality of the Studies Included

Quality assessment using ROBINS-I, RoB 2.0, and the GRADE framework revealed variable certainty across the study designs. Among the 15 observational studies, most were judged to have a moderate risk of bias, primarily due to residual confounding factors, participant selection, and limitations in outcome measurement. Histological and mechanistic studies with small sample sizes [22,23,24,25] have been downgraded to a serious risk of bias, reflecting their limited external validity and heterogeneous sampling. The only randomized controlled trial [28] demonstrated a low risk of bias in most domains, although some concerns related to deviations from the intended interventions led to an overall moderate certainty of evidence. Genetic evidence derived from Mendelian randomization [34] was rated with moderate certainty, supported by large-scale GWAS datasets and consistency across analyses. However, some indirectness was noted due to restrictions on European ancestry populations. The overall certainty of the evidence was low to moderate, supporting an association between immune dysregulation and atrial fibrillation, while highlighting the need for larger, multicenter, high-quality, prospective studies, as shown in Supplementary Materials.

## 4. Discussion

This synthesis combines findings from histological, mechanistic, interventional, prognostic, and genetic studies, encompassing data from over 50,000 patients and more than one million individuals included in Mendelian randomization analyses. Collectively, these studies support the conclusion that immune dysregulation is a key contributor to the pathogenesis and outcomes of AF. Evidence spans from regional atrial infiltration by T lymphocytes and macrophages to systemic immune imbalances involving circulating immune cell subsets and genetically determined immune traits.

A recurring theme across these levels of evidence is T cell-driven inflammation, which emerges as a central mechanism linking immune activation to atrial remodeling and clinical complications. Immune disparity—particularly in T cell subsets—consistently appears as an independent predictor of outcomes, regardless of traditional risk stratification tools.

Several immune biomarkers have demonstrated prognostic significance: soluble CD40 ligand (sCD40L) predicts thromboembolic complications in chronic AF (Duygu et al. [20]); CD4^+^CD28^−^ T cells independently predict cardiovascular mortality in patients with AF and heart failure (Sulzgruber et al. [21]); and aberrant CD40/CD40L axis signaling is associated with increased thrombotic risk (Ogurkova et al. [19]). Furthermore, integrative bioinformatic analyses have revealed immune infiltration signatures and dysregulated pathways, including calcineurin–NFAT signaling and reduced regulatory T cell function, that may offer novel therapeutic targets (Xiao et al. [36]).

### 4.1. Immune Cells Infiltration in the Atrium Myocardium

The present findings reinforce prior histological evidence of immune cell infiltration in the atria of patients with AF. In animal models, tachyarrhythmia-induced atrial remodeling has been linked to the infiltration of CD3^+^ T lymphocytes into atrial tissue [19]. In human studies, Yang et al. [37] observed an increased presence of CD68^+^ macrophages and dendritic cells in atrial appendages of patients with AF compared to those in sinus rhythm—findings consistent with those reported by Smorodinova and Yamashita, as included in our review.

Additional mechanistic insight is provided by Vyas et al. [25], who demonstrated that EAT harbors clonally expanded tissue-resident memory T (TRM) cells. These findings introduce a novel immunological dimension to EAT’s role in AF and support clinical observations linking increased EAT volume to AF burden and recurrence following catheter ablation [22,24,25].

### 4.2. Mechanistic Pathways

Beyond descriptive evidence of inflammatory infiltration, emerging mechanistic insights point to critical interactions between immunological, coagulative, and metabolic pathways in AF. Our synthesis highlights that CD8^+^ T cells activated via protease-activated receptor 1 (PAR1) serve as functional mediators linking the coagulation cascade to atrial fibrosis. This finding aligns with work by Yamashita et al. [24], who reported that thrombin enhances T cell migration and promotes the release of proinflammatory cytokines within atrial tissue [22].

In parallel, dysregulation of immune checkpoint pathways—particularly downregulation of PD-1 on T cells and PD-L1 on antigen-presenting cells—mirrors mechanisms observed in autoimmunity and cancer, where impaired checkpoint control leads to sustained T cell activation [23]. These mechanistic observations support a model in which AF persistence is driven not only by electrophysiological remodeling but also by aberrant immune regulation and immuno-coagulative signaling.

### 4.3. Immune Differences Between AF Subtypes

Many studies show that immunological dysregulation worsens from paroxysmal to persistent and chronic AF, suggesting a transition from transient inflammatory activation to lasting structural remodeling. In chronic and persistent AF, histological studies reveal greater infiltration of CD3^+^ and CD68^+^ immune cells, as well as fibrosis and atrial dilatation, compared to paroxysmal variants [21]. Circulating biomarkers reveal a steady increase in pro-inflammatory cytokines (IL-6, IL-17, TNF-α) and a decrease in regulatory T-cell fractions in non-paroxysmal atrial fibrillation. Mechanistic studies have demonstrated that persistent atrial fibrillation (AF) is linked to senescent CD8^+^ T cells, checkpoint failure (PD-1/PD-L1), chronic immunological exhaustion, and tissue remodeling [38]. These findings suggest that paroxysmal atrial fibrillation represents transient immune activation, whereas persistent and chronic atrial fibrillation represent advanced, self-sustaining immunological remodeling. Immune signatures may signal disease burden and AF risk, emphasizing the need for subtype-specific biomarker validation and therapeutic targeting [20].

### 4.4. Ablation–Mediated Immune Response

Our review underscores that catheter ablation is not immunologically inert. Specifically, radiofrequency (RF) ablation has been shown to mobilize progenitor cells, while cryoballoon (CB) ablation is associated with a more favorable long-term immune biomarker profile. These findings align with broader evidence on systemic immune responses following cardiac interventions. Pang et al. [34] reported transient elevations in proinflammatory cytokines, including interleukin-6 (IL-6) and tumor necrosis factor-alpha (TNF-α), after RF ablation. More recently, Liu et al. (2023) demonstrated that CB ablation induces significantly less systemic inflammatory activation [39]. These observations support the notion that immunological consequences should be considered when selecting and evaluating ablation strategies in atrial fibrillation.

### 4.5. Prognostic Utility of Immune Biomarkers

Several studies included in this review demonstrate the prognostic value of specific immune cell subsets in atrial fibrillation (AF). Senescent CD8^+^ T cells (CD8^++^sen) have been identified as independent predictors of stroke and cardiovascular mortality. At the same time, imbalances in the CD4/CD8 ratio are independently associated with both the presence of AF and adverse clinical outcomes. Notably, Zhou et al. [30] reported increased risk with low CD4/CD8 ratios, whereas You et al. [31] observed a similar association with high ratios. This apparent paradox likely reflects underlying differences in patient populations—stable coronary artery disease outpatients in Zhou et al. versus multimorbid inpatients in You et al. A recent study by Chammartin et al. [40] supports the interpretation that both abnormally low and high CD4/CD8 ratios may signal heightened cardiovascular risk under different clinical contexts [26].

Additionally, alterations in regulatory T cell (Treg) populations and dysregulated cytokine signatures—particularly elevated interleukin-6 (IL-6) and interleukin-10 (IL-10)—have consistently been linked to increased stroke risk and AF recurrence [27,28].

### 4.6. Genetic Evidence

A Mendelian randomization study by Feng et al. [34], published in this issue, reported causal associations between immune cell populations and the risk of atrial fibrillation (AF). Specifically, genetically elevated neutrophil, basophil, and CD4^+^ T cell counts were associated with an increased risk of AF. In contrast, higher natural killer (NK) cell and total lymphocyte counts were associated with a protective effect. These findings reinforce prior genome-wide association studies that have implicated immune-related loci, such as the human leukocyte antigen (HLA) region, in AF susceptibility [29]. More recent large-scale MR analyses further validate these findings. You et al. [41] conducted a comprehensive MR study of 731 immunophenotypes, confirming that multiple immune traits—particularly involving T and myeloid cell lineages—have causal effects on AF risk. Notably, the reverse MR analyses in that study helped rule out reverse causality, thereby strengthening the argument that immune dysregulation plays a primary role in disease initiation. Additionally, Huang et al. [42] explored interactions between immune cells and plasma metabolites, revealing complex immune-metabolic networks that influence AF pathogenesis. These findings align with earlier experimental studies in LQT1 animal models, where NK cell depletion exacerbated susceptibility to atrial arrhythmias [30]. This growing body of genetic, immunophenotypic, and mechanistic evidence supports the conclusion that immune dysfunction is not a secondary feature but a central contributor to AF pathophysiology.

### 4.7. Clinical Implications

#### 4.7.1. Risk Stratification Guided by Biomarkers

Our study supports the incorporation of immune biomarkers into clinical risk stratification for atrial fibrillation (AF). Current tools, such as the CHA_2_DS_2_-VASc score, have limited sensitivity in predicting stroke and other adverse outcomes, particularly in borderline or low-risk populations. Immune markers—including senescent CD8^+^ T cells, the CD4/CD8 ratio, and systemic inflammation scores—demonstrate additional prognostic value beyond conventional clinical variables. Prospective external validation in large, multicenter cohorts is warranted to establish the utility of these models in guiding treatment decisions, particularly regarding anticoagulation and rhythm-control strategies.

#### 4.7.2. Anticipated Targets

The identification of immune pathways involved in atrial fibrillation (AF) opens avenues for novel therapeutic strategies. Targeted inhibition of CD8^+^ T cell signaling via protease-activated receptor 1 (PAR1) may attenuate thromboinflammatory responses, while modulation of immune checkpoints—such as restoring PD-1/PD-L1 signaling—could re-establish immune tolerance. Additionally, strategies aimed at reducing epicardial adipose tissue (EAT) volume or altering its immunologic profile may lower AF burden. These approaches align with a growing interest in anti-inflammatory therapies for cardiovascular disease, exemplified by the CANTOS (canakinumab) and COLCOT (colchicine) trials [43,44,45,46,47]. However, translating these immunomodulatory interventions into AF management will require rigorous clinical evaluation to ensure safety, efficacy, and appropriate patient selection.

#### 4.7.3. Procedural Approaches

The observation that cryoballoon (CB) ablation leads to a more favorable biomarker trajectory compared to radiofrequency (RF) ablation suggests that the immune response plays a meaningful role in mediating procedural outcomes in AF. For example, Zhou et al. [48] found that RF ablation produces greater elevations in inflammatory markers and myocardial injury biomarkers than cryoballoon ablation. Similarly, Boano et al. [49] reported higher peaks of myocardial injury and cell stress markers (e.g., troponin T, CK-MB, PAR-1, HSP27) after cryothermia (cryoballoon) versus RF when used in the setting of mitral valve surgery; notably, the markers of classic inflammation like CRP, IL-6, and IL-18 did not differ significantly between modalities, reflecting that different components of the immune response are variably affected by ablation energy source. Osmancik et al. [50] also demonstrated that RF tends to elicit a stronger proinflammatory response, whereas CB induces less sustained inflammatory activation over time.

These findings suggest that immune profiling—measuring both acute biomarker spikes (injury, coagulation, stress) and the longer-term inflammatory response—could be considered as secondary endpoints in ablation trials. Doing so may help identify phenotypes of patients who respond better to CB versus RF in terms of recurrence, adverse remodeling, or rhythm outcomes. Such stratification could guide procedure choice, post-procedure management (e.g., anti-inflammatory therapy), and improve overall personalization of AF ablation strategies.

#### 4.7.4. Implementation and Cost-Effectiveness Factors

Immune biomarkers must be biologically validated, cost-effective, scalable, and integrated into established treatment pathways to treat AF. Electrophysiology practitioners rarely utilize immunological assays, such as multiparametric flow cytometry or cytokine profiling, because they are resource-intensive and non-standardized. Research suggests that biomarker-guided strategies will only be sustainable if they offer a significant advantage over traditional clinical scores, such as CHA-DS-VASc or ABC-AF risk models, especially in terms of cost per QALY and healthcare system capacity [51,52]. Integrating immunological measures into clinical processes requires standardized phenotyping techniques, automated data pipelines, and electronic decision support systems [53]. To ensure feasibility, reimbursement, and regulatory compliance, implementation science frameworks emphasize multidisciplinary collaboration among electrophysiology, immunology, and health economics [54].

Thus, while immune biomarkers have great potential for precision risk stratification and targeted immunomodulatory therapy in AF, their adoption will depend on rigorous clinical impact, cost-effectiveness, and real-world applicability in integrated cardiovascular care models [55].

### 4.8. Limitations

Several limitations of the available evidence and this systematic review should be noted, with the first being the heterogeneity of study designs. The included studies were histological investigations, mechanistic translational studies, interventional cohorts, prognostic registries, and Mendelian randomization. Such diversity contributes to the scope of coverage but impedes direct comparison, thereby precluding quantitative synthesis via meta-analysis. Second, patient selection bias. Most histological and translational studies have been performed in heavily selected surgical populations with advanced valvular or structural heart diseases. These results cannot be extrapolated to patients with early non-valvular or paroxysmal AF. In addition, the prognostic cohorts ranged from outpatients with coronary artery disease to inpatients with multiple comorbidities, which may have affected the way of the association (e.g., CD4/CD8 ratio). Third, our sample size was small. Some mechanistic and histological studies included fewer than 50 patients, which weakened not only the statistical power but also the subgroup analysis. This results in an increased risk of errors in the first and second types. Fourth, no uniform immune phenotypes were observed. The studies included various panels (e.g., CD3/CD68 immunohistochemistry versus flow cytometry for CD4/CD8 ratios vs. senescence or checkpoint markers). The lack of interchangeability between standardized definitions and assays hinders the comparison and merging, fifth, geographical, and ancestry restrictions. Most studies have been conducted in European and East Asian populations. In addition to methodological limitations, the Mendelian randomization study was conducted using GWAS data from populations of European ancestry only, thereby restricting its applicability to other populations [34]. The risk of bias in most studies was rated as moderate to severe, primarily due to confounding factors, selection bias, and the presence of publication bias; relatively few studies adjusted for comorbidities or medications that could alter immune signatures.

Finally, due to methodological and clinical heterogeneity, meta-analysis was not conducted after systematic identification and critical assessment. Histological case–control, mechanistic translational, prognostic, and Mendelian randomization studies reported different immunological biomarkers and outcomes. Variability in assay methodologies, immunophenotyping panels, sample time (tissue vs. circulation), and outcome measures (AF recurrence, fibrosis load, or inflammatory indices) prevented effective statistical pooling without breaching homogeneity and independence assumptions. Furthermore, several papers lacked standardized effect estimates (such as odds ratios, hazard ratios, or mean differences) suitable for meta-analysis. Quantitative synthesis may become possible as the area progresses. Subgroup-specific meta-analyses could evaluate T-cell subsets and cytokine levels as predictors of AF recurrence or immune checkpoint expression across AF subtypes, utilizing harmonized immune biomarker data reporting, standardized measurement platforms, and consistent outcome definitions. Quantitative effect estimates, meta-regression by research type, and causal inference would result from such analyses. For now, qualitative evidence synthesis remains the most effective approach for integrating mechanistic, clinical, and genetic findings into a translational framework [56,57,58].

### 4.9. Future Directions

Longitudinal immune profiling throughout the atrial fibrillation (AF) continuum is crucial for elucidating how immune dysregulation evolves. Recent multi-omics studies, which integrate transcriptomics, metabolomics, and epigenetics with immune cell phenotyping, have begun to map temporal changes in immune cell composition and function in disease and aging. For example, Gong et al. [59] used longitudinal multi-omics immune profiling. They found that T cells accumulate age-related transcriptional changes more than other immune cells, even in the absence of overt inflammation or pathology, suggesting a baseline of immunosenescence that may predispose to arrhythmic risk. Li et al. [60] performed multi-omics on middle-aged cohorts and demonstrated dynamic regulation of B and T cell antigen receptor signaling and metabolism across time, which may signal the onset of AF or other cardiovascular endpoints. Meanwhile, Conte et al. [61] and others have emphasized the role of EAT as not only an inflammatory depot but also one whose immune profile, adipokines, and metabolic crosstalk may be directly modifiable, thereby impacting the burden and recurrence of AF after ablation.

Recent evidence suggests that innate immune activation, particularly through neutrophil-derived damage-associated molecular patterns such as S100A8/A9, may amplify fibroinflammatory remodeling in AF. This complex acts as both a biomarker and effector of sterile inflammation, linking oxidative stress and atrial structural remodeling. Its translational potential in AF warrants prospective validation [62].

Translationally, these insights open the door for future interventional trials in which AF is included as a primary or secondary outcome of treatments targeting immune or metabolic pathways—such as PAR1 antagonists, EAT-focused therapies, or senolytics. To move toward clinical application, large, prospectively collected, multi-ethnic cohorts with serial immune biomarker sampling are needed to validate and refine risk models. Investigation of immunosenescence—both as a biomarker and therapeutic target (e.g., via immune rejuvenation or senolytic approaches)—also holds promise for modifying AF trajectory rather than just managing symptoms.

Future efforts should prioritize the establishment of standardized immune phenotyping frameworks integrating histological, cytometric, and transcriptomic methodologies. Cross-platform calibration using reference panels and unified antibody clones will be essential to ensure reproducibility and comparability across AF cohorts, ultimately enabling reliable meta-analytic synthesis.

## 5. Conclusions

This systematic review reveals that immune dysregulation is a key factor in the pathogenesis, progression, and outcome of AF. In 16 studies exhibiting histological evidence, local infiltration of T-lymphocytes and macrophages into the atrial myocardium, particularly the left atrium, was observed, accompanied by hypertrophy and fibrosis. Mechanistic investigations have revealed disease-causing immune axes, including CD8+ T cells activated by PAR1 signaling, dysregulation of the inhibitory PD-1/PD-L1 checkpoint, and tissue-resident memory T cells originating from the epicardial adipose tissue. Interventional data have demonstrated the mobilization of progenitor cells and modulation of thromboinflammatory biomarkers in ablated procedures. In prognostic analyses, immune-based signatures (e.g., CD4/CD8 ratio imbalance, senescent CD8+ cells, and regulatory lymphocyte subsets) were consistently identified as independent predictors of worse outcomes, regardless of the established risk scores. Lastly, Mendelian randomization strengthened the evidence for the causal role of neutrophils, basophils, and CD4+T cells in AF and identified natural killer cells as protective factors.

Collectively, these observations support a gradient from local immune infiltration to systemic dysregulation and genetic susceptibility, highlighting the immune context as both a biomarker and therapeutic target. Prospective validation of immune markers in multicenter cohorts is warranted, and immune-modulating strategies, combined with rhythm control and stroke prevention, should be further pursued.

## Figures and Tables

**Table 1:** Baseline characteristics of the included studies.

Study	Sample Size	Age, Years ^†^	Males, *n* (%)	CHA2DS2-VASc ^†^	Hypertension, *n* (%)	Diabetes, *n* (%)	Stroke/TIA, *n* (%)	CAD, *n* (%)	HF, *n* (%)	BMI (kg/m^2^)	LAD (mm) ^†^	LVEF (%) ^†^	Notes
Duygu, et al. [20]	44	58 ± 6	20 (45.5)	CHADS_2_: 0–1	9 (20)	Excluded	Excluded	Excluded	Excluded	NR	45 ± 8	65 ± 5	NVAF; sCD40L
Kim et al. [27]	56 (46 RFCA; 10 EPS)	53.0 ± 13.5	39 (70)	NR	NR	NR	NR	Excluded structural	NR	NR	42.2 ± 7.2	~53–55	RFCA vs. EPS; all failed ≥1 AAD
Yamashita et al. [24]	27 (21 PeAF; 6 PAF)	59.9 ± 10.1	17 (63)	NR	NR	NR	NR	NR	NR	NR	55.2 ± 11.0	63.4 ± 9.2	Maze + valve surgery
Smorodinova, et al. [22]	46 (19 LSPeAF; 27 SR)	AF older by ~7 yrs	NR	NR	Similar	Similar	NR	SR > AF	Similar	NR	LA vol ↑ in AF	NR	Histology; atrial tissue
Sulzgruber, et al. [21]	112 (56 AF; 56 non-AF)	AF: 66.6; non-AF: 61.4	AF: 44 (78.6)	NR	AF: 71.4	AF: 42.9	NR	50.0	100% (HF)	AF: 29.1	NR	EF stratified	Chronic HF cohort
Bin Waleed et al. [28]	58 (29 CB; 29 RF)	CB: 61.2; RF: 62.4	16–18 (≈68)	CB: 1.5; RF: 1.0	CB: 50; RF: 58	CB: 12.5; RF: 7.7	CB: 17.2; RF: 6.9	NR	NR	CB: 25.0; RF: 25.8	CB: 36.5; RF: 36.0	59 (both)	Paroxysmal AF; CB vs. RF
Hohmann, et al. [23]	10	68–73	50–67	NR	NR	NR	NR	Valvular CAD	NR	NR	LA area ↑ in AF	53–62	LAA tissue; inflammatory markers
Li, et al. [33]	210 stroke cases + 87 controls	~63 vs. ~62	55–58	NR	68.6	33.3	9.5 (AF prevalence)	4.8	NR	NR	NR	NR	Ischemic stroke cohort
Xiao, et al. [35]	Public datasets	NR	NR	NR	NR	NR	NR	NR	NR	NR	NR	NR	Bioinformatics only
Ogurkova, et al. [19]	60 AF + 22 controls	60–66	G1: 21; G2: 11	NR	76–81	NR	NR	64–76	NYHA II–III	NR	NR	NR	With/without thrombotic events
Chang, et al. [32]	90 (45 AF; 45 controls)	AF: 63.3; Ctrl: 64.2	58–62	NR	Excluded severe HF	Excluded	Excluded	Excluded	Excluded	AF: 23.5	NR	NR	PD-1/PD-L1; case–control
Friebel, et al. [26]	100 FDAF; 20 controls	AF: 70.1; Ctrl: 67.8	AF: 62.5; Ctrl: 50	AF: 3.98; Ctrl: 3.45	AF: 87.5	AF: 30	AF: 10	NR	AF: 26	AF: 27.6	NR	AF: 59	FDAF cohort; ↑ NT-proBNP
Feng, et al. [34] (MR)	GWAS (~1 M)	N/A	N/A	N/A	N/A	N/A	N/A	N/A	N/A	N/A	N/A	N/A	Mendelian randomization
Zhou, et al. [30]	158 (71 AF; 87 non-AF)	AF: 65.3; non-AF: 47.3	AF: 49; non-AF: 59	NR	AF: 54.9	AF: 56.3	AF: 5.6	AF: 16.9	AF: 11.3	AF: 24.7	AF: 40.7	NR	Catheter ablation cohort
Vyas, et al. [25]	153 (18 AF; 26 SR baseline)	AF: 67.3; SR: 65.6	AF: 78; SR: 65	NR	AF: 67	AF: 11	NR	MI: 17–27	NR	26.6	AF: 4.9 cm; SR: 3.6 cm	AF: 59; SR: 60	Surgical cohort
You, et al. [31]	45,905 (818 AF; 45,087 non-AF; 560 FU)	AF: 72.9; non-AF: 58.6	AF: 58.3; non-AF: 44.0	NR	AF: 62.5	AF: 19.9	20.4 (subcohort)	AF: 13.1	AF: 24.8	24.0	~44–45	~59	Hospital cohort, China

^†^ Values are presented as mean ± standard deviation (SD), median (interquartile range), or as reported in the original study. NR = not reported; NVAF = non-valvular atrial fibrillation; RFCA = radiofrequency catheter ablation; EPS = electrophysiological study; PAF = paroxysmal atrial fibrillation; PeAF = persistent atrial fibrillation; LSPeAF = long-standing persistent atrial fibrillation; SR = sinus rhythm; LA = left atrium; LAA = left atrial appendage; CB = cryoballoon; RF = radiofrequency; AAD = antiarrhythmic drug; FDAF = first-diagnosis atrial fibrillation; GWAS = genome-wide association study; MR = Mendelian randomization; FU = follow-up; CAD = coronary artery disease; HF = heart failure; BMI = body mass index; LAD = left atrial diameter; LVEF = left ventricular ejection fraction; MI = myocardial infarction; NYHA = New York Heart Association functional class. Arrow: ↑ (increase).

**Table 2:** Main findings of selected studies ↑.

Study	Type of Study	Objectives	Main Findings (Aligned with PICOT)
Duygu, et al. [20]	Observational (clinical)	Evaluate soluble CD40 ligand in NVAF	Elevated sCD40L levels in NVAF patients correlated with atrial size, suggesting immune activation contributes to AF persistence and progression.
Kim et al. [27]	Mechanistic (ablation cohort)	Assess atrial remodeling in paroxysmal vs. persistent AF	Structural atrial remodeling (↑ LAD, ↓ LVEF) and immune activation are more pronounced in persistent AF, linking T-cell-mediated changes with disease chronicity.
Yamashita et al. [24]	Histological (surgical)	Evaluate atrial tissue remodeling in valve surgery + Maze	AF patients undergoing Maze surgery exhibited more atrial fibrosis and inflammatory infiltration, supporting immune-driven atrial remodeling.
Smorodinova et al. [22]	Histological (surgical)	Characterize atrial tissue in persistent AF vs. SR	Long-standing AF tissue contained increased T cells and macrophages with enhanced fibrosis, confirming chronic immune activation in AF pathogenesis.
Sulzgruber et al. [21]	Prospective observational (HF cohort)	Assess immune/inflammatory predictors in HF with AF	In HF patients, AF was associated with higher NT-proBNP and immune markers, predicting adverse outcomes beyond standard clinical risk scores.
Bin Waleed et al. [28]	Randomized trial (procedural)	Compare cryoballoon vs. RF ablation in PAF	Ablation techniques (cryoballoon vs. RF) differentially modulated inflammatory markers, showing procedural impact on thrombo-inflammatory pathways in AF.
Hohmann et al. [23]	Mechanistic (surgical)	Analyze LAA tissue and immune pathways	AF atrial appendage tissue exhibited ↑ inflammatory infiltrates and cytokine expression, linking local immune response to AF persistence.
Li et al. [32]	Prospective observational (stroke cohort)	Investigate lymphocyte subsets after acute ischemic stroke	Altered Treg/Th17 balance predicted adverse outcomes in stroke patients, with AF subgroups showing distinct immunological profiles associated with prognosis.
Xiao et al. [36]	Bioinformatics (multi-dataset)	Identify immune-related genes in AF	Bioinformatics integration identified immune-related genes and T-cell activation pathways strongly associated with AF onset and progression.
Ogurkova, et al. [19]	Observational (clinical + immunology)	Assess immune cells in AF with/without thrombosis	AF patients with thrombosis showed greater T-cell activation and PD-1 dysregulation, linking immune exhaustion with prothrombotic risk in AF.
Chang et al. [32]	Case–control (immunology)	Evaluate PD-1/PD-L1 pathway in AF	AF patients exhibited impaired PD-1/PD-L1 signaling, highlighting checkpoint dysregulation as a mechanism for sustained immune activation.
Friebel et al. [26]	Prospective observational (FDAF cohort)	Assess immune markers in newly diagnosed AF	Senescent CD8+ T cells and ↑ NT-proBNP were independent predictors of AF incidence, strengthening immune markers for risk stratification.
Feng et al. [34] (MR)	Genetic (Mendelian randomization)	Explore the causal effects of immune cells on AF	Genetic evidence confirmed causal effects of neutrophils, basophils, and CD4+ T cells in AF risk, with NK cells protective, supporting causality.
Zhou et al. [30]	Prospective cohort (ablation)	Examine the role of senescent CD8+ T cells	Senescent CD8+ T cells predicted AF recurrence after ablation, independent of CHA_2_DS_2_-VASc, underscoring their prognostic utility.
Vyas et al. [25]	Translational (surgical + tissue)	Study epicardial adipose tissue–immune crosstalk	Epicardial adipose tissue–resident T cells produced IL-17 and IFN-γ, promoting atrial fibrosis and AF progression through immune–metabolic crosstalk.
You et al. [31]	Retrospective cohort (hospital-based)	Evaluate the prognostic role of the CDR score in AF	The CDR immune score outperformed CHA_2_DS_2_-VASc in predicting AF-related outcomes, supporting immune-based prognostic stratification.

Abbreviations: NVAF = non-valvular atrial fibrillation; PAF = paroxysmal atrial fibrillation; SR = sinus rhythm; LAA = left atrial appendage; HF = heart failure; RF = radiofrequency; FDAF = first-diagnosis AF; MR = Mendelian randomization; LAD = left atrial diameter; LVEF = left ventricular ejection fraction. Arrows: ↑ represents increase and ↓ represents reduction.

**Table 3:** Immune Predictors of Atrial Fibrillation Outcomes Across Observational and Genetic Studies.

Study	Immune Predictor	Outcomes
Duygu et al. [20]	Soluble CD40 ligand	Thromboembolic risk
Sulzgruber et al. [21]	CD4^+^CD28^−^ T cells; Regulatory T cells	Cardiovascular mortality
Friebel et al. [26]	Senescent CD8^+^ T cells (CD28^−^CD57^+^)	Stroke, heart failure, hospitalization, cardiovascular death
Zhou et al. [30], You et al. [31]	Imbalance of CD4/CD8 ratio	Adverse cardiovascular outcomes
Ogurkova et al. [19]	CD40/CD40L axis	Thrombotic complications
Xiao et al. [36]	Reduced Tregs, increased myeloid infiltration	Persistent AF
Feng et al. [34]	Genetically elevated neutrophil, basophil, and CD4^+^ T cell counts; higher NK cells and total lymphocytes	Increased risk (first group); protection (second group)

Abbreviations: AF—Atrial Fibrillation; Tregs—Regulatory T Cells; CD—Cluster of Differentiation; NK cells—Natural Killer Cells; CD57^+^—Surface markers used to characterize T cell phenotypes.

## Data Availability

The raw data supporting the conclusions of this article will be made available by the authors upon request.

## Supplementary Materials

The following supporting information can be downloaded at https://www.mdpi.com/article/10.3390/jcm14207316/s1, Methods S1: PRISMA Checklist; Methods S2: PRISMA Abstract Checklist; Methods S3: Search Strategy; Results S1: Risk of Bias–Robins I and Robs 2; Results S2: Grade Evidence Profile (per study); Results S3: Summary of Findings (SoF) by Outcome Domain. Reference [28] is cited in Supplementary Materials.

## Abbreviations

The following abbreviations are used in this manuscript:

AF	Atrial fibrillation
SR	Sinus rhythm
LA	Left atrium/left atrial
LAA	Left atrial appendage
EAT	Epicardial adipose tissue
TRM	Tissue-resident memory T cells
PD-1	Programmed cell death protein-1
PD-L1	Programmed death-ligand 1
PAR1	Protease-activated receptor-1
MMP-9	Matrix metalloproteinase-9
GROβ	Growth-related oncogene-β
CB	Cryoballoon ablation
RF	Radiofrequency ablation
RFCA	Radiofrequency catheter ablation
CDR	CD4/CD8 ratio
Tregs	Regulatory T cells
Bregs	Regulatory B cells
DNTs	Double-negative T cells
SIS	Systemic inflammation score
CHA_2_DS_2_-VASc	Clinical risk score for stroke in AF
HF	Heart failure
MACE	Major adverse cardiovascular events
MR	Mendelian randomization
GWAS	Genome-wide association study
RoB	Risk of bias
GRADE	Grading of Recommendations Assessment, Development, and Evaluation
n	sample size
HR	Hazard ratio
OR	Odds ratio
CI	Confidence interval
