# Supplementary material for: From Tissue to Transcriptome: A Systematic Review of Multi-Level Evidence for Immune Dysregulation in Atrial Fibrillation
Source: J Clin Med. 2025 Oct 16;14(20):7316. doi: 10.3390/jcm14207316 (PMC12565675; doi:10.3390/jcm14207316)
Supplement: Supplementary file 1 [file jcm-14-07316-s001.zip › jcm-3918168-supplementary.pdf]

From Tissue to Transcriptome: A Systematic Review of Multi-Level Evidence for Immune Dysregulation in Atrial Fibrillation

Antônio da Silva Menezes Junior, M.D., Ph.D.,[1,2], Isabela Jubé Wastowski[3], BSc, Ph.D., Henrique Lima de Oliveira, MS[1], Khissya Beatryz Alves de Lima[1], Silvia Marçal Botelho[1,2], M.D. PhD

[1]Internal Medicine, Faculty of Medicine, Federal University of Goiás, Goiânia, Brazil

[2]Pontifical Catholic University of Goiás, Life and Medical Sciences School, Internal Medicine, Goiânia, Brazil

[3]Statal University of Goiás, Molecular Immunology Department, Goiânia, Brazil.

Address for correspondence:

Antonio da Silva Menezes Junior, M.D., PhD, FESC

Address: 5ª Avenida, s/n - Setor Leste Universitário, Goiânia - GO, 74605-050

Office number: +5562982711177

Email: a.menezes.junior@uol.com.br

Summary

Supplementary Methods S1. PRISMA Checklist:.. 3

Supplementary Methods S2. PRISMA Abstract Checklist 5

Supplementary Methods S3. Search Strategy 7

Supplementary Results S1. Risk of Bias – Robins I and Robs 2:.. 8

Supplementary Results S2 -Grade Evidence Profile (per study):………………………………………………………………………..…………………... ……….
…10

Supplementary Results S3 -Summary of Findings (SoF) by Outcome Domain **..** 12

Footnotes:... 12

Supplemental Methods S1. PRISMA Checklist:

Section and Topic	Item #	Checklist item	Location where item is reported
**TITLE**			
Title	1	Identify the report as a systematic review.	*p.1*
**ABSTRACT**			
Abstract	2	See the PRISMA 2020 for Abstracts checklist.	*p.2*
**INTRODUCTION**			
Rationale	3	Describe the rationale for the review in the context of existing knowledge.	*p.3-4*
Objectives	4	Provide an explicit statement of the objective(s) or question(s) the review addresses.	*p.4*
**METHODS**			
Eligibility criteria	5	Specify the inclusion and exclusion criteria for the review and how studies were grouped for the syntheses.	*p.5*
Information sources	6	Specify all databases, registers, websites, organisations, reference lists and other sources searched or consulted to identify studies. Specify the date when each source was last searched or consulted.	*p.6*
Search strategy	7	Present the full search strategies for all databases, registers and websites, including any filters and limits used.	*p.6-7*
Selection process	8	Specify the methods used to decide whether a study met the inclusion criteria of the review, including how many reviewers screened each record and each report retrieved, whether they worked independently, and if applicable, details of automation tools used in the process.	*p.6-7*
Data collection process	9	Specify the methods used to collect data from reports, including how many reviewers collected data from each report, whether they worked independently, any processes for obtaining or confirming data from study investigators, and if applicable, details of automation tools used in the process.	*p.6-7*
Data items	10a	List and define all outcomes for which data were sought. Specify whether all results that were compatible with each outcome domain in each study were sought (e.g. for all measures, time points, analyses), and if not, the methods used to decide which results to collect.	*p.7-8*
	10b	List and define all other variables for which data were sought (e.g. participant and intervention characteristics, funding sources). Describe any assumptions made about any missing or unclear information.	*p.7-8*
Study risk of bias assessment	11	Specify the methods used to assess risk of bias in the included studies, including details of the tool(s) used, how many reviewers assessed each study and whether they worked independently, and if applicable, details of automation tools used in the process.	*p.8-9*
Effect measures	12	Specify for each outcome the effect measure(s) (e.g. risk ratio, mean difference) used in the synthesis or presentation of results.	*p.9*
Synthesis methods	13a	Describe the processes used to decide which studies were eligible for each synthesis (e.g. tabulating the study intervention characteristics and comparing against the planned groups for each synthesis (item #5)).	*p.9*
	13b	Describe any methods required to prepare the data for presentation or synthesis, such as handling of missing summary statistics, or data conversions.	*p.9*

Section and Topic	Item #	Checklist item	Location where item is reported
	13c	Describe any methods used to tabulate or visually display results of individual studies and syntheses.	*p.9*
	13d	Describe any methods used to synthesize results and provide a rationale for the choice(s). If meta-analysis was performed, describe the model(s), method(s) to identify the presence and extent of statistical heterogeneity, and software package(s) used.	*p.9*
	13e	Describe any methods used to explore possible causes of heterogeneity among study results (e.g. subgroup analysis, meta-regression).	*p.9*
	13f	Describe any sensitivity analyses conducted to assess robustness of the synthesized results.	*p.9*
Reporting bias assessment	14	Describe any methods used to assess risk of bias due to missing results in a synthesis (arising from reporting biases).	*p.9 At* Supplemental Results 3
Certainty assessment	15	Describe any methods used to assess certainty (or confidence) in the body of evidence for an outcome.	*p.9 At* Supplemental Results 3-4
**RESULTS**			
Study selection	16a	Describe the results of the search and selection process, from the number of records identified in the search to the number of studies included in the review, ideally using a flow diagram.	*p.10 at Figure 1*
	16b	Cite studies that might appear to meet the inclusion criteria, but which were excluded, and explain why they were excluded.	*p.10*
Study characteristics	17	Cite each included study and present its characteristics.	*p.10-11*
Risk of bias in studies	18	Present assessments of risk of bias for each included study.	*p.10-11*
Results of individual studies	19	For all outcomes, present, for each study: (a) summary statistics for each group (where appropriate) and (b) an effect estimate and its precision (e.g. confidence/credible interval), ideally using structured tables or plots.	*p.11-17*
Results of syntheses	20a	For each synthesis, briefly summarise the characteristics and risk of bias among contributing studies.	*p.17-19*
	20b	Present results of all statistical syntheses conducted. If meta-analysis was done, present for each the summary estimate and its precision (e.g. confidence/credible interval) and measures of statistical heterogeneity. If comparing groups, describe the direction of the effect.	*p.17-19*
	20c	Present results of all investigations of possible causes of heterogeneity among study results.	*p.17-19*
	20d	Present results of all sensitivity analyses conducted to assess the robustness of the synthesized results.	*p.17-19*
Reporting biases	21	Present assessments of risk of bias due to missing results (arising from reporting biases) for each synthesis assessed.	*p.18-19 at* Supplemental Methods 2
Certainty of evidence	22	Present assessments of certainty (or confidence) in the body of evidence for each outcome assessed.	*p.18-19*

Section and Topic	Item #	Checklist item	Location where item is reported
**DISCUSSION**			
Discussion	23a	Provide a general interpretation of the results in the context of other evidence.	*p.20-25*
	23b	Discuss any limitations of the evidence included in the review.	*p.24-25*
	23c	Discuss any limitations of the review processes used.	*p.24-25*
	23d	Discuss implications of the results for practice, policy, and future research.	*p.25-26*
**OTHER INFORMATION**			
Registration and protocol	24a	Provide registration information for the review, including register name and registration number, or state that the review was not registered.	*p.6*
	24b	Indicate where the review protocol can be accessed, or state that a protocol was not prepared.	*p.6* PROSPERO: CRD 420251038380
	24c	Describe and explain any amendments to information provided at registration or in the protocol.	NA
Support	25	Describe sources of financial or non-financial support for the review, and the role of the funders or sponsors in the review.	*p.2*
Competing interests	26	Declare any competing interests of review authors.	*p.2*
Availability of data, code, and other materials	27	Report which of the following are publicly available and where they can be found: template data collection forms; data extracted from included studies; data used for all analyses; analytic code; any other materials used in the review.	MS

Supplementary Methods S2. PRISMA Abstract Checklist

Section and Topic	Item #	Checklist item	Reported (Yes/No)
**TITLE**			
Title	1	Identify the report as a systematic review.	*p.1*
**BACKGROUND**			
Objectives	2	Provide an explicit statement of the main objective(s) or question(s) the review addresses.	*p.1-2*
**METHODS**			
Eligibility criteria	3	Specify the inclusion and exclusion criteria for the review.	*p.2*

Section and Topic	Item #	Checklist item	Reported (Yes/No)
Information sources	4	Specify the information sources (e.g. databases, registers) used to identify studies and the date when each was last searched.	*p.2*
Risk of bias	5	Specify the methods used to assess risk of bias in the included studies.	*p.2*
Synthesis of results	6	Specify the methods used to present and synthesise results.	*p.2*
**RESULTS**			
Included studies	7	Give the total number of included studies and participants and summarise relevant characteristics of studies.	*p.2*
Synthesis of results	8	Present results for main outcomes, preferably indicating the number of included studies and participants for each. If meta-analysis was done, report the summary estimate and confidence/credible interval. If comparing groups, indicate the direction of the effect (i.e. which group is favoured).	*p.2-3*
**DISCUSSION**			
Limitations of evidence	9	Provide a brief summary of the limitations of the evidence included in the review (e.g. study risk of bias, inconsistency and imprecision).	*p.3*
Interpretation	10	Provide a general interpretation of the results and important implications.	*p.3*
**OTHER**			
Funding	11	Specify the primary source of funding for the review.	NA
Registration	12	Provide the register name and registration number.	NA

Supplemental Materials Methods S3– Search Strategy

Database	Search Strategy
**PubMed (MEDLINE)**	("Atrial Fibrillation"[Mesh] OR "atrial fibrillation"[tiab] OR AF[tiab]) AND ("T-Lymphocytes"[Mesh] OR "T cell*"[tiab] OR "regulatory T cell*"[tiab] OR Treg*[tiab] OR CD4[tiab] OR CD8[tiab] OR "PD-1"[tiab] OR PD1[tiab] OR "PD-L1"[tiab] OR PDL1[tiab] OR Interleukin*[tiab] OR Cytokine*[tiab] OR "immune biomarker*"[tiab] OR "immune dysregulation"[tiab] OR Senescen*[tiab]) AND (diagnos*[tiab] OR prognos*[tiab] OR predict*[tiab] OR "risk stratification"[tiab] OR recurren*[tiab] OR progression[tiab]) NOT (animals[MeSH Terms] NOT humans[MeSH Terms])
**Embase (Elsevier/Ovid)**	('atrial fibrillation'/exp OR 'atrial fibrillation':ti,ab OR AF:ti,ab) AND ('t lymphocyte'/exp OR 'regulatory t lymphocyte'/exp OR 'cd4 lymphocyte'/exp OR 'cd8 lymphocyte'/exp OR 'programmed cell death 1 receptor'/exp OR 'programmed death ligand 1'/exp OR interleukin/exp OR cytokine/exp OR 'immune biomarker':ti,ab OR 'immune dysregulation':ti,ab OR 't cell*':ti,ab OR treg*:ti,ab OR cd4:ti,ab OR cd8:ti,ab OR 'pd-1':ti,ab OR 'pd-l1':ti,ab OR senescen*:ti,ab) AND (diagnos*:ti,ab OR prognos*:ti,ab OR predict*:ti,ab OR 'risk stratification':ti,ab OR recurren*:ti,ab OR progression:ti,ab) NOT ('animal'/exp NOT 'human'/exp) AND [2000-2025]/py
**Scopus**	TITLE-ABS-KEY("atrial fibrillation" OR AF) AND TITLE-ABS-KEY("T cell*" OR "regulatory T cell*" OR Treg* OR CD4 OR CD8 OR "PD-1" OR PD1 OR "PD-L1" OR PDL1 OR Interleukin* OR Cytokine* OR "immune biomarker*" OR "immune dysregulation" OR Senescen*) AND TITLE-ABS-KEY(diagnos* OR prognos* OR predict* OR "risk stratification" OR recurren* OR progression) AND PUBYEAR > 1999 AND PUBYEAR < 2026 AND NOT (TITLE-ABS-KEY(animal*) AND NOT TITLE-ABS-KEY(human*))

Supplementary Results S1. Risk of Bias – Robins 1

Study	Bias due to confounding	Bias in the selection of participants	Bias in the classification of interventions	Bias due to deviations from intended interventions	Bias due to missing data	Bias in the measurement of outcomes	Bias in the selection of the reported result	Overall risk of bias judgement
Conradi 2004	Serious	Serious	Moderate	Low	Low	Low	Moderate	Serious
Kim, 2009	Serious	Serious	Moderate	Low	Moderate	Moderate	Moderate	Serious
Yamashita,2015	Serious	Serious	Moderate	Low	Low	Low	Moderate	Serious
Smorodinova,2017	Serious	Serious	Moderate	Low	Low	Low	Moderate	Serious
Bin Waleed,2019	Moderate	Serious	Moderate	Low	Low	Low	Moderate	Serious
Hohmann, 2020	Serious	Serious	Moderate	Low	Moderate	Low	Moderate	Moderate
Li, 2021	Moderate	Moderate	Low	Low	Moderate	Moderate	Moderate	Serious
Chang, 2022	Serious	Serious	Moderate	Low	Moderate	Low	Moderate	Serious
Friebel,2023	Serious	Serious	Moderate	Low	Low	Low	Moderate	Serious
Vyas,2024	Serious	Serious	Moderate	Low	Low	Low	Moderate	Moderate
Stegbauer,2024	Moderate	Moderate	Low	Low	Low	Low	Moderate	Moderate
Zhou,2024	Moderate	Moderate	Low	Low	Serious	Low	Moderate	Moderate
You, 2025	Serious	Serious	Low	Low	Moderate	Moderate	Moderate	Serious

Supplementary Results S1. Risk of bias summary for randomized studies (RoB 2)

**Study**	**Bias from randomization process**	**Bias due to deviations from intended interventions**	**Bias due to missing outcome data**	**Bias in the measurement of the outcomes**	**Bias in the selection of the reported result**	**Overall risk of bias**
Bin Waleed, et al. [28]	Some concerns	Low	Low	Low	Some concerns	Low

Supplementary Results S2. Grade Evidence Profile (per study):

Diagnostic/Prognostic systematic review of immune biomarkers in atrial fibrillation (AF). Certainty judgments follow GRADE (DTA/prognosis narrative) per individual study.

Study	Design	Index test/biomarker focus	Key outcome(s)	Risk of bias	Inconsistency	Indirectness	Imprecision	Publication bias	Overall certainty
**Duygu, 2008**	Observational (clinical)	sCD40L	Association with AF; relation to LA size	Serious (selection/confounding)	NA (single)	Moderate (association only)	Serious (small N)	Unclear	Very low (⊕◯◯◯)
**Kim, 2009**	Mechanistic (ablation cohort)	Remodeling + immune activation (surrogates)	AF persistence/chronicity	Moderate	NA (single)	Moderate (procedural cohort)	Serious (small/modest N)	Unclear	Low (⊕⊕◯◯)
**Yamashita, 2015**	Histology (surgical)	Tissue immune infiltration/fibrosis	AF presence (surgical subset)	Serious (selection/measurement)	NA	Critical (tissue-only)	Serious	Unclear	Very low (⊕◯◯◯)
**Smorodinova, 2017**	Histology (surgical)	T cells/macrophages, fibrosis	AF presence (long-standing)	Serious	NA	Critical	Serious	Unclear	Very low (⊕◯◯◯)
**Sulzgruber, 2017**	Prospective observational (HF)	Inflammatory markers (clinical)	AF association, outcomes in HF	Moderate	NA	Moderate (HF cohort)	Moderate	Unclear	Moderate–Low (⊕⊕◯◯)
**Bin Waleed, 2019**	Randomized (PAF ablation)	Procedural effect on inflammatory markers	Periprocedural changes; technique differences	Low–Some concerns	NA	Moderate (procedural setting)	Moderate (n=58)	Unclear	Moderate (⊕⊕◯◯)
**Hohmann, 2020**	Mechanistic (surgical)	LAA inflammatory infiltrate/cytokines	AF presence/severity	Serious	NA	Critical (tissue-only)	Serious	Unclear	Very low (⊕◯◯◯)

**Li, 2021**	Prospective cohort (stroke)	Treg/Th17 balance	Long-term outcomes; AF subgroup differences	Moderate–Serious	NA	Serious (stroke population)	Serious	Unclear	Very low (⊕◯◯◯)
**Xiao, 2021**	Bioinformatics	Immune-related gene signatures	Association with AF (in silico)	Serious (indirect data)	NA	Critical (no clinical testing)	Serious	Unclear	Very low (⊕◯◯◯)
**Ogurkova, 2022**	Observational (clinical+immunology)	T-cell activation; PD-1 pathway	Thrombosis in AF; immune exhaustion	Moderate	NA	Moderate (mixed AF phenotypes)	Serious (small N)	Unclear	Low (⊕⊕◯◯)
**Chang, 2022**	Case–control	PD-1/PD-L1 signaling	AF vs control discrimination; activity	Moderate–Serious (spectrum bias)	NA	Serious (case–control)	Serious	Unclear	Very low–Low (⊕/⊕⊕◯◯)
**Friebel, 2023**	Prospective observational (FDAF)	Senescent CD8+; NT-proBNP	Incident AF; risk stratification	Moderate	NA	Not serious (clinical cohort)	Moderate	Unclear	Low (⊕⊕◯◯)
**Feng, 2023**	Mendelian randomization	Immune cell counts (GWAS)	Causal effect on AF risk	Low–Moderate	Low	Moderate (ancestry; genetic proxy)	Low	Low	Moderate (⊕⊕⊕◯)
**Zhou, 2024**	Prospective cohort (ablation)	Senescent CD8+ T cells	Post-ablation AF recurrence	Moderate	NA	Not serious (ablation cohort)	Moderate (events, CI)	Unclear	Low (⊕⊕◯◯)

**Vyas, 2024**	Translational (surgical+tissue)	EAT resident T cells; IL-17/IFN-γ	AF progression (mechanistic)	Serious	NA	Critical (tissue-heavy)	Serious	Unclear	Very low (⊕◯◯◯)
**You, 2025**	Retrospective cohort (+ FU)	CDR immune score vs CHA$_2$DS$_2$-VASc	Prognosis; composite outcomes	Moderate	NA	Not serious	Not serious–Moderate	Unclear	Low–Moderate (⊕⊕◯◯)
**Kazem, 2020**	Observational (surgical POAF)	Inflammation/comorbid burden	POAF occurrence	Moderate	NA	Moderate (surgical)	Moderate	Unclear	Low (⊕⊕◯◯)

Supplementary Results S3 -Summary of Findings (SoF) by Outcome Domain:

Outcome (index test)	No. studies (design)	Certainty (GRADE)	Direction of effect	Main reasons for rating down
**Senescent $CD8^+$ (diagnosis & post-ablation recurrence)**	2 (obs. cohorts)	**Low (⊕⊕◯◯)**	↑ Levels in AF: predict recurrence	Risk of bias; imprecision
**PD-1/PD-L1 pathway markers (diagnosis/activity)**	2 (case–control)	**Very low (⊕◯◯◯)**	Impaired checkpoint signaling in AF	Risk of bias; indirectness; imprecision
**Treg/Th17 balance (prognosis)**	1 (stroke cohort)	**Very low (⊕◯◯◯)**	Altered balance predicts worse outcomes; AF subgroup distinct	Indirectness; imprecision
**CDR immune score vs CHA$_2$DS$_2$-VASc (prognosis)**	1 (large cohort)	**Low–Moderate (⊕⊕◯◯)**	**Outperforms CHA$_2$DS$_2$-VASc**	Single-study body; unclear publication bias
**sCD40L (association with AF)**	1 (small observ.)	**Very low (⊕◯◯◯)**	Higher in AF; correlates with LA size	Risk of bias; imprecision

**Histologic immune infiltration (tissue)**	4 (surgical tissue)	**Very low (⊕◯◯◯)**	Consistent immune–fibrotic signal	Indirectness; risk of bias
**Genetic MR (immune traits → AF risk)**	1 (MR)	**Moderate (⊕⊕⊕◯)**	Neutrophils/basophils/$CD4^{+}$ ↑ risk; NK protective	Indirectness (ancestry; not a test)

Footnotes:

1. Risk of bias: Many studies are case-control or single-center cohorts; histologic studies are small and selective.

2. Indirectness: Specialized cohorts (surgical, stroke); tissue/genetic findings not direct diagnostics.

3. Inconsistency: Generally consistent effects; heterogeneity in thresholds and platforms.

4. Imprecision: Small samples, wide CIs, lack of external validation.

5. Publication bias: Unclear; few studies per biomarker; selective reporting possible.
